# Awake Craniotomy in Patients With Language Deficits: A Retrospective Cohort Study in 48 Patients

**DOI:** 10.1227/ons.0000000000001806

**Published:** 2025-10-22

**Authors:** Suzanne L. Hartung, Irene M. C. Huenges Wajer, Martine J. E. van Zandvoort, Pierre A. Robe

**Affiliations:** * Department of Neurology and Neurosurgery, University Medical Center Utrecht, Utrecht, the Netherlands;; ‡ Current affiliation: Department of Medical Psychology, Amsterdam University Medical Center, Amsterdam, the Netherlands;; § Department of Experimental Psychology, Helmholtz Institute, Utrecht University, Utrecht, the Netherlands

**Keywords:** Awake craniotomy, Language, Cognitive monitoring, Feasibility

## Abstract

**BACKGROUND AND OBJECTIVES:**

This retrospective cohort study reviewed the feasibility and applicability of awake surgery for glioma in patients with clinically relevant preoperative language deficits, specifically focusing on intraoperative language monitoring and its impact on postoperative language outcomes.

**METHODS:**

Group and individual analyses were conducted to compare preoperative and postoperative language deficits. In addition, an overview of intraoperative tests was provided, and an evaluation of the monitoring process was performed.

**RESULTS:**

In 100% of patients with significant (z score ≤ −1 SD) preoperative language deficits (N = 48), language tasks could be performed during awake surgery. In 90% of these cases, functional boundaries for language function were found during surgery.

**CONCLUSION:**

This study confirms the feasibility and applicability of monitoring language functions during awake brain surgery when preoperative language deficits are present. Both low-grade and high-grade glioma patients show an overall stable performance on a language comprehension test and an improved performance on a language production test 3 to 6 months after surgery and, therefore, seemed to benefit from cognitive monitoring.

**ABBREVIATIONS:**
BNT: Boston Naming Test
TT: Token Test.

In the past decades, awake brain surgery has become the preferred surgical technique in patients suffering from a brain tumor in eloquent areas.^1,2^ Peroperative cognitive monitoring helps to preserve language and other cognitive functions and serves to maintain the patient's quality of life, while maximizing surgical outcomes in both patients with low- and high-grade gliomas.^3^ In our University Medical Center, this approach has been systematically applied since January 2011, leading to an increase in the number of awake craniotomies performed. This policy has notably improved of return-to-work rates and longer active careers for patients.^4^

Several studies have provided language protocols that can be used during awake surgery.^2,5,6^ A recent systematic review^7^ showed the value of intraoperative testing on a cortical and subcortical level and provides an overview on language localization. As complex as language itself is, it is apparent that many different pathways and regions are involved. Cortically, widespread locations across the frontal, parietal, temporal, and occipital lobes are linked to different speech and language errors during peroperative electrical stimulations, with the most errors occurring in the precentral gyrus. Subcortically, mostly the inferior fronto-occipital fascicule but also the frontal aslant tract and the arcuate, superior longitudinal, inferior longitudinal, and uncinate fascicle are associated with speech and language errors,^7^ potentially confirming the multiplicity of pathways and possible abundance of pathways. It underscores the need to administer both cortical and subcortical mapping of language function.

Although the benefits of awake craniotomies support its integration into modern surgical standards, patient selection remains a nuanced process. Decision making in neurosurgery does not only depend on factors such as the medical condition, age, and comorbidity of the patient. It is also influenced by the expectations of both the patient and the neuro-oncological team regarding benefits and risks of the procedure.^8^ Another critical consideration is the feasibility of intraoperative monitoring, particularly in patients with preoperative language deficits. Several cases have been described as failed awake surgery attributed to preoperative language deficits^9,10^ As a result, these deficits have often been seen as a potential challenge for effective intraoperative monitoring, leading some studies to exclude such patients from consideration^11^ Notwithstanding the fact that communication is essential to perform awake surgery and intraoperative monitoring, it is debatable whether the presence of preoperative language deficits per definition rules out intraoperative monitoring. This is also evident from recent case reports that have demonstrated positive outcomes after awake surgery in glioma patients with preoperative language deficits, both in respect to cognitive outcome and maximized resection.^12,13^ This suggests that these deficits do not necessarily preclude successful intraoperative monitoring or favorable cognitive outcomes.

At our center, we consider language deficits in decision making without treating them as an automatic contraindication. This approach is grounded in 3 key reasons: First, language and its components are primordial functions that allow the patients to communicate and/or understand with their environment—as such, it is a condicio sine qua non of participation to subsequent treatments and of quality of life. Second, and especially in the case of malignant gliomas, deficits often improve during surgery as the mass effect of the tumor is progressively relieved. Third, even in patients with severe language deficits, it is often possible to compare baseline language functions and use nonverbal tests to assess specific language aspects and identify the (sub-)cortical structures involved. This allows for effective intraoperative monitoring.

Since 2011, we have systematically considered patients with preoperative language deficits in awake surgeries. This study shares our experience with this approach and evaluates the application of intraoperative language monitoring when preoperative language deficits are present. We aim to (1) provide an overview of language tests that can be administered intraoperatively in the presence of preoperative language deficits and (2) assess whether language functions could indeed be safeguarded or improved 3-6 months after surgery.

## METHODS

### Ethics

This study does not fall under the scope of the Dutch Medical Research Involving Human Subjects Act and does not require medical ethics approval. However, an independent quality check confirmed compliance with legislation and regulations regarding informed consent, data management, privacy aspects, and legal aspects (protocol number 23U-0430).

### Patient Characteristics

**FIGURE 1.**
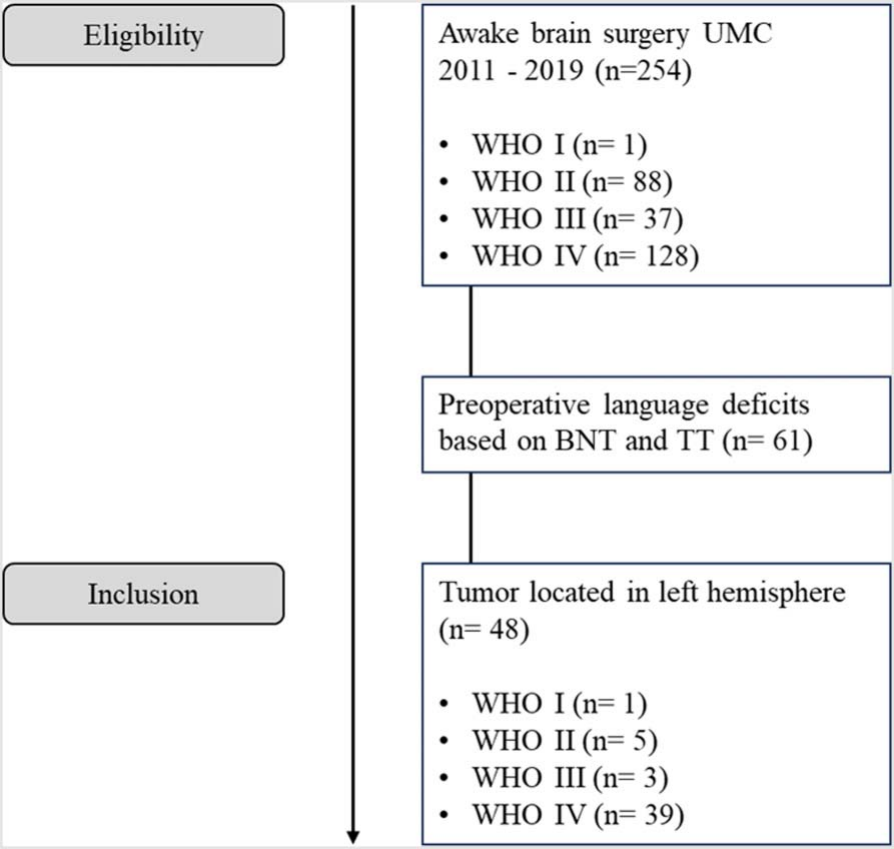
The flowchart of inclusion. BNT, Boston Naming Test; TT, Token Test; UMC, University Medical Center.

We reviewed all adult patients (n = 254) who had undergone a first-time awake craniotomy for low-grade (World Health Organization grade-I/grade-II) or high-grade glioma (World Health Organization grade-III/grade-IV) between February 2011 and July 2019 in our University Medical Center. Inclusion criteria were being at least 18 years old and native Dutch speakers. In addition, the brain tumor needed to be located in the dominant (left) hemisphere and preoperative language deficits needed to be present at preoperative neuropsychological assessment 1–3 days before surgery, based on performances on the Boston Naming Test (BNT) and Token Test (TT). For patient selection, Z-scores on both tests were calculated and at least one of the available tests needed to be affected (ie, ≤−1 SD) to be labeled as preoperative language deficit, to be sensitive to any possible language disturbances. In total, 48 patients met our inclusion criteria, see Figure 1. Two patients were excluded from awake surgery because of their inability to comprehend both the instructions for the awake procedure and the intraoperative test commands, such as following simple verbal orders.

### Pre- and Postoperative Neuropsychological Assessment

A clinical neuropsychologist administered the BNT and TT as part of a concise preoperative neuropsychological assessment, covering the main cognitive domains. This assessment took place 3 to 1 day(s) before surgery. The timing of the postoperative neuropsychological assessment depended on tumor grade. Owing to the progressiveness of high-grade gliomas, patients were seen 3 months after surgery, whereas patients with a low-grade glioma were assessed 6 months after surgery.

The 30-item BNT^14^ was used to assess naming ability. This test consists of 30 black-and-white line drawings of objects, presented in sequence from most common to least common. The patient described in 1 word what is seen on the picture of the object presented, confrontation naming. Scores were calculated according to the guidelines and norms by Van Loon-Vervoorn (2005).^14^

The Revised 21-item TT was used to assess auditory language comprehension. It consists of plastic tokens that vary in shape and color that have to be placed in position by the patient, following verbally given instructions. Instructions of this revised version are a Dutch translation of the series of 5 elements of the TT.^15^ A cutoff score was used to determine deficits, corrected for educational level.

### Direct Electrical Stimulation

Surgeries were conducted fully awake under local anesthesia, using microscope, ultrasound, and neuronavigation guidance. The procedures were performed in a park-bench position, allowing the patients to relax and to face the anesthetist and/or the clinical neuropsychologist in a comfortable manner. The head was fixed in a Mayfield clamp placed under local anesthesia (using a mix of 5 mg/mL Chirocaine 1:1 v:v and 2% lidocaine with adrenaline 1:200 000, a total of 29-55 cc for the complete procedure) injected at the pin sites of the Mayfield clamp and in a rectangular fashion around the planned skin incision site. After removal of the bone flap using a high-speed Anspach® drill, anesthetics mix-soaked gelatin foam was applied onto the dura at the level of the meningeal arteries. Patients also received titrated pain sedation and relaxation with remifentanyl and propofol, respectively. Tumors were approached using standard routes and removed using an ultrasonic aspirator at ultra-low power under constant corticosubcortical stimulations using a Ojeman® cortical stimulator (50 Hz, 1 ms, 2-4 mAmps, trains of 3 seconds at the cortical level and continuous stimulations at 1-2 mAmps at the subcortical level). The stimulation was performed with a bipolar probe, working with the ultrasonic aspirator in the direct vicinity of the stimulation probe. Threshold was only verified at the cortical level: Multiple functions were tested over the exposed surgical cortex—tailored on the anatomic area considered—at increasing amplitudes, starting at 2 mAmps by increments of 0.5 mAmps to a maximum of 4 mAmps. If no function could be found at 4 mAmps, the cortex was considered devoid of testable function and the surgery proceeded to the subcortical level, where standard amplitudes of 2 mAmps were used for the resection, with decrease to 1 mAmp at the places where function was found, to bring the resection as close as safely possible to these areas. Subcortically, the tests were performed continuously under stimulations in trains of 50 Hz. When mistakes or hesitations were made by the patient, those areas were then stimulated repeatedly for a minimum of 4 seconds subcortically or for the minimal duration needed by the patient to perform the test. Areas of stimulation that provoked deficits in the concomitantly performed neuropsychological tests reliably (ie, 3 times) were considered as functional for that test. In addition, subcortical stimulations that provoked phosphenes, even only once, were considered as belonging to the optic radiations and respected. For the cortex, the stimulations were conducted for sequences of 4 seconds, interrupted by slight displacements of the bipolar stimulation probe to avoid inducing seizure activities (using this method, no seizure was induced in any of the patients) when the tests lasted longer than 4 seconds. No patients required conversion to general anesthesia.

### Intraoperative Language Testing

Validated and experimental neuropsychological language tests were used during surgery. Test batteries were individually tailored to meet the patient's need and increase the chances of a feasible assessment. Eleven tests were administered (Table 1), with supplementary information provided on tailored tests in the Supplemental file, http://links.lww.com/ONS/B248.^6,15-17^

**TABLE 1.** Neuropsychological Language Tests Administered During Awake Brain Surgery in Our University Medical Center

Task	Aim of measure	Example
DuLIP—Action naming^6^	Verb production and syntactic processing	Picture of a man who eats cake: “the man …,” answer “eats cake” 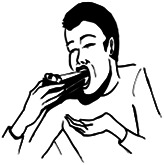
Sentence reading^6^	Semantic comprehension and articulatory processing	“He wears brown shoes”, “How often do you play tennis?”
Repetition of words^6^	Word production	“pilot”, “birthday”, “library”
DuLIP—Naming with semantic odd picture out^6^	Nonverbal semantic judgment and naming	Choose which image is the odd 1 out: 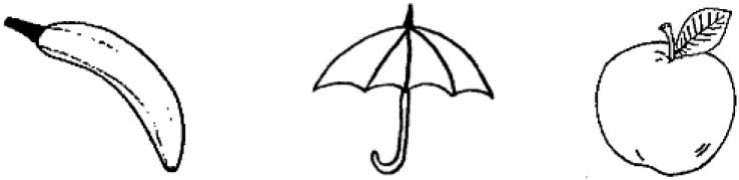
Token Test^15^	Auditory language comprehension	Put the red circle on top op the green rectangular 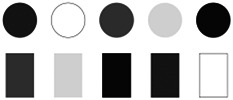
Screeling—word picture pairing^16^	Semantic associations	Choose out of 6 pictures, which 1 pairs with the word written in the middle 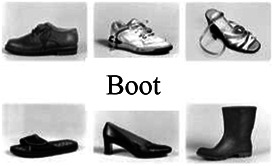
Screeling—syntax^16^	Syntactic processing	Choose which picture pairs with the sentence “the paper lays on top of the book” 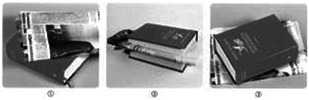
Snodgrass object naming test^17^	Word retrieval	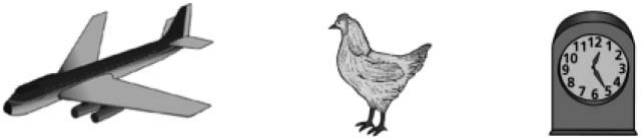
Story reading^a^	Semantic comprehension and articulatory processing	Short stories on facts about nature, animals and cars
Speaking spontaneously^a^	Speech production and picture comprehension	Talking about a topic of choice or explaining the context of a picture of people eating and drinking
Writing on dictation^a^	Writing, organization of text in space, displacements or substitutions of letters and repetitions	“Pencil”, “desk”, “microwave”

a Adaptation described in Supplementary file, http://links.lww.com/ONS/B248.

### Language Model

Speech and language are widely distributed through corticosubcortical networks, and the neuroanatomy of language has been described in various models.^18^ We chose the functional neuroanatomic model of auditory speech processing, proposed by Hickok and Poeppel (2015),^19^ as a theoretical framework. This dual stream model holds that the dorsal stream is essential for speech production, and the ventral stream is involved in language comprehension.^20^ During intraoperative cognitive monitoring, the type of error is documented by the clinical neuropsychologist to gain insight into specific subcortical pathways involved in specific speech and language errors. Intraoperative, functional boundaries are defined according to the standard clinical practice during cognitive monitoring.^21^ To cluster the wide variety of speech and language errors made during surgery, errors are assigned to 1 of 4 categories: motor speech, mental lexicon, language comprehension, and speech production.

### Statistical Analysis

To test possible differences between preoperative and postoperative scores on the BNT and TT, paired samples Wilcoxon signed rank tests were performed, as data were not normally distributed. Given that we tested 2 constructs simultaneously, we applied a Bonferroni correction to adjust for multiple comparisons. In addition, we calculated change index scores and categorized the change into 1 of 3 categories [*improved language abilities, stable language abilities and increase in language deficits*]. Data were analyzed using SPSS (IBM, SPSS Statistics, 29.0.1.0).

## RESULTS

### Preoperative Functional Assessment

**FIGURE 2.**
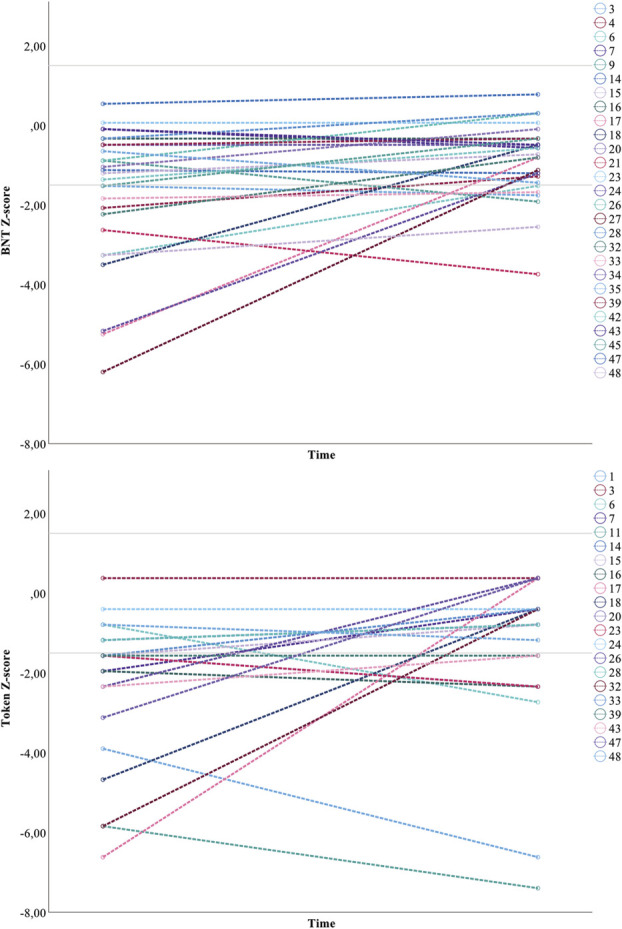
Spaghetti plots depicting individual patient trajectories on the BNT (top panel) and Token Test (bottom panel) before and after surgery. Each line represents 1 patient's performance from the preoperative to the postoperative assessment. The x-axis indicates the time point, and the y-axis shows the Z-scores. Variation in trajectory slopes reflects individual differences in cognitive change after surgery. BNT, Boston Naming Test.

Clinical and demographic study characteristics are summarized in Table 2. Preoperative language assessment revealed deficits in 21 cases based on the TT, 16 cases on the BNT, and 11 cases on both tests. Figure 2 details the severity of these language deficits and change over time. Overall, 27 of 48 patients (56%) exhibited severe language deficits, with z-scores indicating deviations of at least 2 SDs below the mean, with Z-scores ranging from −2 to −6.6. Data on the histological and molecular tumor subtypes are presented in Table 3.

**TABLE 2.** Study Characteristics

	Total	Preoperative language deficits based on both BNT and TT	Preoperative language deficits based on BNT	Preoperative language deficits based on TT
Patients [*n* (%)]	48	11 (22%)	16 (33%)	21(45%)
Sex (M/F)	29/19	8/3	8/8	13/8
Age at surgery [mean (range)]	59 y (21-78)	60 y (43-74)	53 y (21-74)	63 y (40-78)
WHO (n)				
WHO I	1	0	1	0
WHO II	5	0	3	2
WHO III	3	1	1	1
WHO IV	39	10	11	18
Preoperative KPS (range)	86 (50-100)	84 (50-100)	88 (70-100)	84 (60-100)
Epileptic seizure	19/48 (40%)	3/8 (25%)	10/16 (63%)	6/21 (29%)

KPS, Karnofsky Performance Score; WHO, World Health Organization.

**TABLE 3.** Cognitive Outcomes and Change Indices by Histological and Molecular Tumor Subtype

Histological type	IDH status	1p/19q status	N	Postoperative Z-score BNT	Change Index BNT	Postoperative Z-score token	Change Index token
Glioblastoma multiforme	Wildtype	—	38	−0.9 ± 1.0	+0.9 ± 1.7	−1.4 ± 1.8	+1.3 ± 2.5
A2	Mutant	—	5	−1.1 ± 0.6	+1.1 ± 0.9	−1.8 ± 1.4	-0.6 ± 1.9
A3	Mutant	—	1	−0.6^a^	−0.5	−0.4	+1.6
O2	Mutant	1p/19q co-del	1	−0.6^a^	+0.8	n.a	n.a
O3	Mutant	1p/19q co-del	1	−1.8^a^	−0.2	+0.4	0

IDH, isocitrate dehydrogenase; n.a., not assessed.

a Single value, no SD.

The clinical presentation is characterized by patients exhibiting severe word-finding difficulties, inadequate responses to questions during spontaneous speech, and both phonological and semantic errors. Perseveration may occur, with patients resorting to circumlocution when attempting to describe target words.

### Intraoperative Functional Boundaries

**FIGURE 3.**
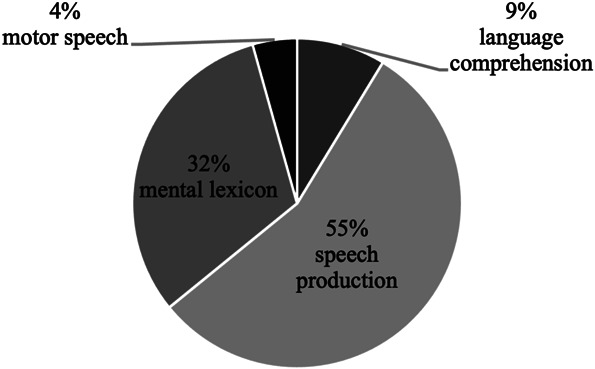
Categorized language errors provoked by direct electrical stimulation during awake surgery based on the model of Hickok and Poeppel.

All 48 patients underwent intraoperative language testing. In 43 patients (90%), functional language boundaries were found, enabling maximally safe functional-based resections. Figure 3 displays the frequency of direct electrical stimulation–provoked disturbances, classified in modalities based on the speech processing model of Hickok and Poeppel (2015).

The severity of preoperative language deficits did not significantly differ between patients with and without detectable functional boundaries. Although a formal statistical comparison of preoperative deficits and the inability to define functional boundaries was not feasible because of sample size limitations, descriptive analysis indicates no clear correlation. Patients with functional boundaries detected had mean Z-scores of −1.79 (BNT) and −2.17 (TT), whereas those without detected boundaries (10%) showed relatively better scores of −1.62 (BNT) and −1.95 (TT).

### Immediate Postoperative Performance (Within 5 Days)

Although formal neuropsychological testing was not performed early postoperatively because of early discharge and the likely influence of fatigue and edema—clinical impressions of language function were routinely documented. Based on multidisciplinary clinical reports, incorporating observations from clinical neuropsychologists, nursing staff, and the neurosurgeon, approximately 60% of patients demonstrated language function consistent with their preoperative baseline, 35% showed improvement, and only 5% exhibited a decline within the first 5 days postsurgery.

### Postoperative Functional Assessment

On group level, a Wilcoxon signed rank test showed significant improvement in BNT performance 3–6 months after awake surgery (*Z* = −2.515, *P* = .012) compared with the preoperative performance on this test, with a moderate effect size (r = 0.484). Performance on the TT did not significantly improve or decline 3-6 months after awake surgery (*Z* = −1.375, *P* = .169). Glioma grade did not affect outcomes.

The change index (individual level) of pre- and postoperative performance on the BNT (n = 27) showed in 30% of patients a significant improvement of language abilities, with scores improved by +1.2 SD up to +5.1 SD. Language abilities remained stable in 63% of patients (−0.79 SD to +0.95 SD), and 7% of patients showed an increase in language deficits (−1.11 SD to −1.03 SD). The change index of pre- and postoperative TT performance (n = 21) showed improved language abilities in 33% of patients, with scores improved by +1.2 SD up to +7 SD. Language abilities were stable in 52% of patients (−0.78 SD to +0.78 SD), and 14% of patients showed more language deficits (−2.72 SD to −1.55 SD).

## DISCUSSION

Our findings demonstrate both the feasibility and the applicability of awake surgery in the presence of preoperative language deficits. It shows to be suitable to test language abilities in all patients with preoperative language deficits included in this study, resulting in finding the functional boundaries in 90%. Above all, language abilities can be preserved and even be improved after 3 to 6 months after surgery by performing cognitive monitoring.

Our results indicate, primarily, that excluding a patient from awake surgery solely based on the factor of preoperative language deficits is neither necessary nor desirable. Even in our sample including severe language deficits, only 2 patients evaluated for awake surgery were deemed unsuitable because of their inability to comprehend instructions. This offers a perspective shift toward what is achievable within the confines of awake surgery and intraoperative monitoring. Of note, we sometimes observed that patients showed clinical improvement in function during surgery as the tumor became debulked, allowing even more testing.

Consistent with Collée and colleagues (2023),^7^ key anatomic structures to consider for language preservation in surgical planning are found both on a cortical level and subcortical level. As language consists of multiple components (ie naming, repetition, reading, writing, syntax), each component can be linked to a specific cortical-subcortical network. The location of these networks varies across individuals, and imaging data before surgical resection do not show the functional connectivity.^18^ Hence, relying on preoperative (diffusion tract) imaging data alone is not sufficient for safeguarding cognition and it is advisable to conduct intraoperative monitoring. To help identify the functional involvement of the inferior fronto-occipital fascicule and arcuate fascicule, several suggestions can be given to consider such as using the DuLIP—naming with semantic odd one out picture test, story reading test, and Snodgrass object naming test. The complex nature of multitudes emphasizes the importance of using different language tests and ongoing tailoring during cognitive monitoring.

The results also indicate that awake surgery can be beneficial in both low-grade and high-grade glioma patients. Even though awake surgery has become a solid option for the resection of low-grade gliomas, it is not equally as often applied to high-grade gliomas, despite its known benefits.^22^ The reserved attitude might be caused by the higher prevalence of preoperative language deficits reported by high-grade glioma patients compared with low-grade glioma patients, also evident in our data. Preservation of language skills and communication is, however, arguably, even more crucial for high-grade glioma patients as it is needed for consenting to and undergoing optimal cytotoxic adjuvant treatment, and there is only limited time for extensive rehabilitation. Bonifazi and colleagues^3^ showed the positive linguistic outcomes after awake surgery in 19 high-grade glioma patients. The current study supports these findings in a larger cohort, as most patients suffered from a high-grade glioma. In addition, dropping the presence of preoperative language deficits as a contraindication for awake surgery would not only be beneficial for postoperative language outcome but also allows to monitor additional cognitive functions such as executive functioning, memory, and psychomotor speed.^2^

### Limitations

A limitation of this study is its primary reliance on clinical practice rather than being designed as a scientific study. Had the study been designed as a research endeavor from the outset, it could have incorporated additional outcome measures and enhanced the depth of theoretical models. Implementing a schematic tool to illustrate the correlation between tests and anatomic features would have been a beneficial enhancement in this regard. Nevertheless, showcasing the dynamic nature of clinical practice provides a nuanced view of integrated research into clinical settings, fostering innovation and enriching both domains with complementary perspectives. Although this study reassessed the standard procedures and confirms the greater applicability of awake surgery, a limited group of patients must still undergo asleep surgery because of contraindications, such as extreme anxiety, allergy to local anesthetics, inability to communicate (verbal or nonverbal), and inability to lie still for a decent amount of time.^23^ It would be insightful to compare cognitive outcomes between awake and asleep surgery patients, using collaborative series of patients and propensity score matching.

## CONCLUSION

The present findings underscore that awake surgery with tailored cognitive monitoring is feasible and effective even in patients with preoperative language deficits. This approach enables precise functional mapping, supports language preservation and postoperative improvement, and challenges the notion of excluding such patients from awake procedures and highlights the value of individualized, intraoperative testing in both low- and high-grade glioma patients.

## Supplementary Material

## SUPPLEMENTAL DIGITAL CONTENT

**Supplemental digital content:** An explanation on the adaptation of intraoperative language tests.
