# Supplementary material for: Awake Craniotomy in Patients With Language Deficits: A Retrospective Cohort Study in 48 Patients
Source: Oper Neurosurg. 2025 Oct 22;31(2):285–92. doi: 10.1227/ons.0000000000001806 (PMC13348829; doi:10.1227/ons.0000000000001806)
Supplement: Supplementary file 1 [file ons-31-285-s001.docx]

**Adaptation of intraoperative language tests**

**Story reading**

The story reading tests used intraoperatively consists of paragraph-long stories at the highest proficiency level based on the Dutch AVI system (Analoog Vloeiend Lezen). This level reflects the reading skills a person should have acquired by the end of primary school.

**Speaking spontaneously**

During the preoperative assessment, the clinical neuropsychologist will discuss various topics with the patient that they can talk about during surgery. These topics are of interest to the patient but do not carry an emotional load (such as talks about family or friends) to avoid emotional interference during the procedure.

**Writing on dictation**

The patient is asked to write words that are dictated, ranging from simple one syllable words to more complex four-syllable words.
